# Baicalein inhibits progression of osteosarcoma cells through inactivation of the Wnt/β-catenin signaling pathway

**DOI:** 10.18632/oncotarget.20987

**Published:** 2017-09-18

**Authors:** Guo Dai, Di Zheng, Qianliang Wang, Jian Yang, Gaiwei Liu, Qi Song, Xiangran Sun, Chunjie Tao, Qingzhu Hu, Tian Gao, Ling Yu, Weichun Guo

**Affiliations:** ^1^ Department of Orthopedics, Renmin Hospital of Wuhan University, Wuhan 430060, Hubei Province, P. R. China; ^2^ Department of Urology Surgery, Renmin Hospital of Wuhan University, Wuhan 430060, Hubei Province, P. R. China; ^3^ Department of Orthopedic Oncology, Key Laboratory of Carcinogenesis and Translational Research, Ministry of Education, Peking University Cancer Hospital & Institute, Beijing 100000, P. R. China

**Keywords:** baicalein, osteosarcoma, proliferation, apoptosis, Wnt signaling pathway

## Abstract

Osteosarcoma is a very common type of malignant bone tumor in children and young adults and aberrant activation of Wnt/β-catenin signaling pathway has been discovered in osteosarcoma. The traditional Chinese medicine baicalein was proved to have anti-proliferative and anti-metastatic properties in osteosarcoma, but the mechanism remained poorly understood. In the present study, we assessed the effects of baicalein on osteosarcoma and detected the potential molecular mechanism. We found that baicalein significantly suppressed the proliferation of osteosarcoma cells in a concentration- and time-dependent manner. In additional, baicalein could induce apoptosis and cell cycle arrest and reduce cell motility. Moreover, the level of β-catenin and its target genes, including c-myc, cyclinD1, and survivin significantly decreased in baicalein-treated osteosarcoma cells, whereas exogenous expression of β-catenin could reverse the anti-proliferative and anti-metastatic effects of baicalein. Subsequently, we established a 143B xenograft tumor model and found that baicalein treatment significantly inhibited tumor growth accompanied with inhibiting Wnt/β-catenin pathway. Thus, these findings suggest that baicalein may be a potentially effective Chinese herbal medicine for therapeutics of osteosarcoma and Wnt/β-catenin signaling pathway may serve as an efficient molecular marker or predictive target for osteosarcoma.

## INTRODUCTION

Osteosarcoma is a very common type of malignant bone tumor in children and young adults that is marked by high rate of recurrence and early metastasis. It often located in the long bone including the distal femur and proximal tibia [1]. The treatments of osteosarcoma patients mainly include surgery and neoadjuvant chemotherapy. However the use of chemotherapeutic agents, such as doxorubicin and cisplatin, are always along with the high risk of short term and long term adverse effects, such as cardiotoxicity and nephrotoxicity, whereas the outcome is still undesirable [2]. Despite with the development of diagnosis and treatment of osteosarcoma, the prognosis is still not very good and the 5-year survival rate of patients with lung metastasis is less than 20% [3]. The tumor recurrence, metastasis and drug resistance have always been a knotty problem for osteosarcoma treatment, which creates the bottleneck for capture of osteosarcoma [4]. This might be due to the non-specific targeting of anticancer drugs towards the cancer cells [5]. Hence, new drugs specifically targeting the tumor cells should be explored.

The Wnt/β-catenin signaling pathway plays an important role in modulating proliferation, survival, differentiation, metastasis and maintaining stem cell properties of cancer cells [6–8]. It works when the Wnt ligands bind with the receptors which induce the relase of β-catenin. Then the β-catenin moves to nucleus and activates a range of downstream target genes including c-myc, cyclin D1 and survivin. Aberrant activation of Wnt/β-catenin signaling pathway was discovered in many tumors including osteosarcoma [8–10]. Whereas inhibition of Wnt/β-catenin signaling had been proved to suppress osteosarcoma cell survival and growth and inhibit the formation of osteosarcoma stem cells *in vitro* and *in vivo* [11–15].

**Figure 1:**
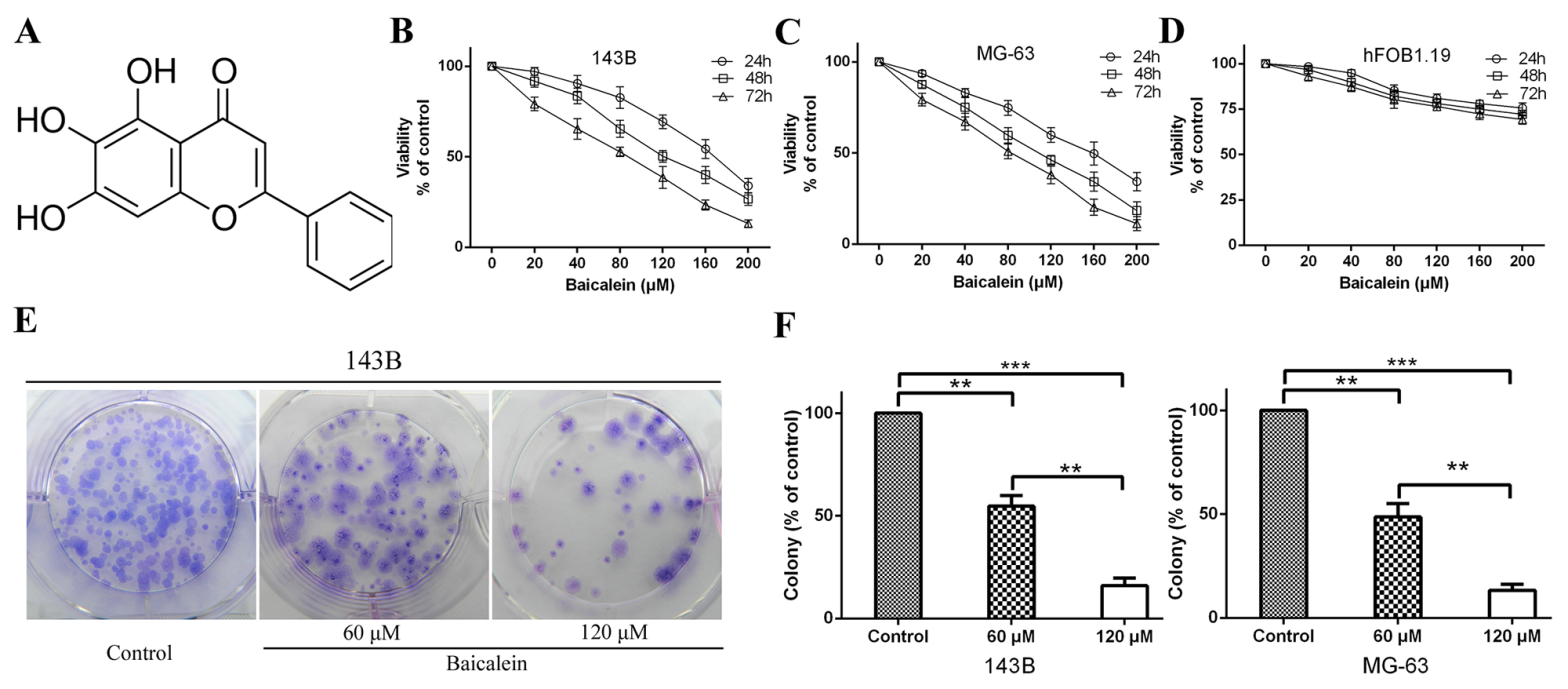
Baicalein inhibits osteosarcoma cells proliferation *in vitro* **(A)** Molecular structure of baicalein. The molecular formula of baicalein is C_15_H_10_O_5_ and its molecular weight is 270.24. **(B** and **C)** 143B and MG-63 cells were incubated with increasing doses of baicalein (20–200 μM) for 24, 48 and 72 h. CCK-8 assay was performed to determine the cytotoxic effect of baicalein. Each point represents means ± SD of three independent experiments. **(D)** Normal human osteoblast cells were incubated with increasing doses of baicalein (20–200 μM) for , 24, 48 and 72 h and analyzed for cell proliferation. Each point represents means ± SD of three separate experiments. **(E)** Representative images from the colony-formation assay. Osteosarcoma 143B cells were incubated with or without baicalein (60 and 120 μM) for 24 h and allowed to grow into colonies for 14 d. **(F)** Quantitative analysis of colony forming rate of 143B and MG-63 cells. Each bar represents means ± SD of three separate experiments. **p < 0.01, ***p < 0.001.

In recent decades, studies have shown that a number of traditional Chinese medicine have potential chemotherapies for osteosarcoma such as cinobufagin, oridonin, sinomenine and so on [16–19]. Baicalein (Figure 1A) is a herbal medicine derived from the root of *Scutellaria baicalensis Georgi* [20, 21]. Many researchers have carried out a relatively thorough study of the anticancer effects of Baicalein. Kim *et al.* [22] found that baicalein could prevent CT26 colon cancer cell metastasis to the lung *in vivo* due to its anti-platelet effects, which mediated through the inhibition of ERK2, p38, and Akt phosphorylation along with activation of PKA-dependent VASP phosphorylation. Ma *et al.* [23] demonstrated that baicalein markedly inhibited the proliferation, migration, and invasion of breast carcinoma cell line MDA-MB-231 *in vitro*. And their results of assays which were carried out in xenograft nude mouse model also exhibited an inhibitive effect of baicalein on tumor metastasis *in vivo*. It was possibly by inhibition of EMT (epithelial-mesenchymal transition), which may be attributed to both down-regulation of SATB1 and Wnt/β-catenin signaling pathway. Therefore, we speculated whether baicalein could inhibit the Wnt/β-catenin pathway to exert anti-proliferation and anti-metastasis in osteosarcoma cells as well.

Herein, the aim of our present study was to confirm the effects of baicalein on the Wnt/β-catenin signaling pathway and its ability to suppress progression of osteosarcoma cells. To this end, we treated the 143B and MG-63 cells with baicalein and then dissected the possible mechanisms. We found for the first time that baicalein could exert anti-proliferation and anti-metastasis effect on osteosarcoma both *in vitro* and *in vivo*, which were partly through inhibiting the Wnt/β-catenin pathway.

## RESULTS

### Baicalein inhibits osteosarcoma cells proliferation *in vitro*

In order to identify the anti-proliferation effect of baicalein, six different concentrations of baicalein (20, 40, 80, 120, 160 and 200 μM) were used to treat the 143B and MG-63 cells for 24, 48 and 72 h. Then, the CCK-8 assay was employed to prove the anti-proliferation effect of baicalein. As shown in Figure 1B and 1C, the proliferation of 143B and MG-63 cells were significantly inhibited by baicalein. Moreover, they showed a concentration- and time-dependent manner. To detect whether baicalein could inhibit normal human osteoblast cell growth, we used hFOB1.19 cells as a control. The CCK-8 assay showed that baicalein treatment resulted in little proliferation reduction of hFOB1.19 cells (Figure 1D). These results showed that baicalein could relative specifically inhibit osteosarcoma cell growth and survival. The IC_50_ (half-maximal inhibitory concentration) values of baicalein in 143B and MG-63 were ∼100-120 μM after 48 h treatment (Table 1). To investigate the potential molecular mechanism of baicalein induced inhibition of osteosarcoma cells progress, we selected 60 and 120 μM baicalein for subsequent cytology experiments *in vitro*. The colony formation assay is a very effective method to detect the long-term proliferation capacity of single cell. Therefore, we carried out clonogenic assays to further investigate the anti-proliferation effect of baicalein on 143B and MG-63 cells growth. Compared to the control group, cellular colony formation was markedly reduced with the treatment of baicalein. It is worth noting that high concentration of baicalein could lead to an even higher percentage of reduction than the low concentration of agent (Figure 1E and 1F). All these results indicate that baicalein could suppress the proliferation of osteosarcoma cells.

**Table 1:** IC_50_ of baicalein in osteosarcoma cell lines

Cell lines	IC_50_ (μM)		
	Baicalein		
	24 h	48 h	72 h
143B	170.1 ± 5.3	118.5 ± 3.5	85.6 ± 2.9
MG-63	160.2 ± 6.2	105.4 ± 3.1	80.6 ± 2.6

Cells were treated with varying concentrations of Baicalein (20 - 200 μM) for 24, 48 and 72 h. The growth inhibitory effect of Baicalein on osteosarcoma cells were evaluated by CCK-8 assay. IC_50_ values shown here are the means ± SD from the data of three independent experiments.

### Baicalein alters cell cycle of osteosarcoma cells

**Figure 2:**
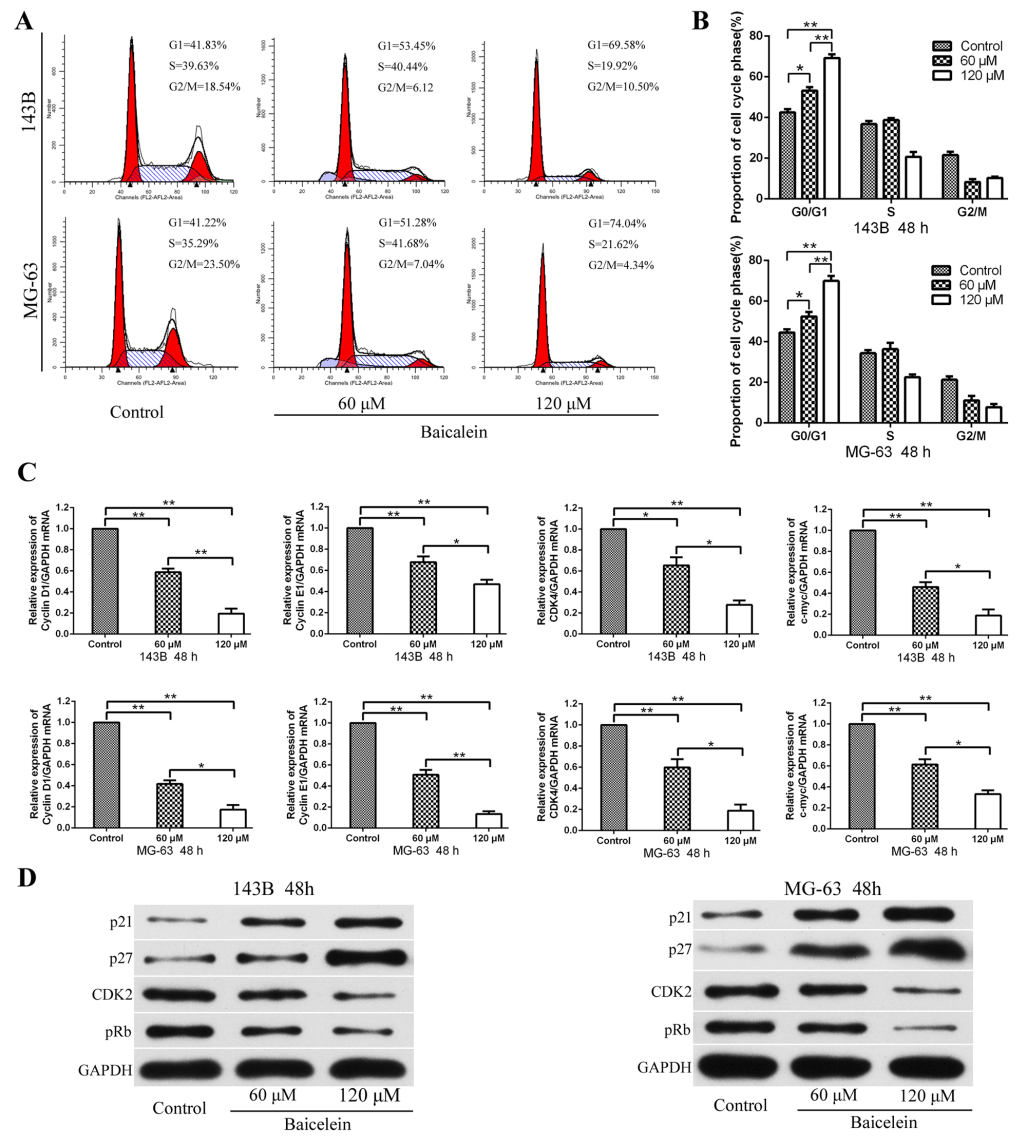
Baicalein induces G1 phase arrest of osteosarcoma cells **(A)** Flow cytometry histograms of osteosarcoma cell DNA content distribution in each phase after treatment with baicale at the indicated concentrations for 48 h showing G1-phase arrest. **(B)** Quantitative analysis percentage of cells distributed in each phase of the cell cycle. **(C)** After treatment as in A, total RNA were extracted from cultured osteosarcoma cells and probed with specific primers. Representative results of Cyclin D1, Cyclin E1, CDK4, and c-myc mRNA levels were as determined by qRT-PCR analysis. **(D)** After treatment as in A, proteins were extracted from osteosarcoma cells and probed with appropriate dilutions of specific antibodies. Representative results of p21, p27, CDK2, and pRb protein levels were as determined by a Western blot analysis; GAPDH was used as the internal control. All data are expressed as the mean ± SD of three independent experiments. *P < 0.05, **p < 0.01, ***p < 0.001.

According to previous reports, cell growth restriction is often associated with cell cycle arrest [24, 25]. In order to determine whether baicalein could impact the cell cycle distribution of 143B and MG-63 cells, the flow cytometry analysis was conducted. As shown in Figure 2A and 2B, the percentage of cells in the G1-phase increased markedly after treatment with 60 and 120 μM baicalein for 48 h, which exhibited in a concentration dependent manner. It meaned that baicalein could cause G1-phase cell cycle arrest in osteosarcoma cells. Subsequently, in order to identify the potential molecular mechanisms that baicalein induced G1-phase arrest, the expressions of cell cycle related mRNA and proteins in response to baicalein treatment were investigated. Firstly, the transcription of genes related to the cell cycle were examined. RT-qPCR revealed that baicalein significantly blocked the genes involved in transcriptional acceleration during the cell cycle, including Cyclin D1, Cyclin E1, CDK4, and c-myc (Figure 2C). Then we examined p21, p27, CDK2, and pRb proteins expression by Western blot. Baicalein treatment markedly increased the levels of p21 and p27 and decreased the expression of CDK2 and pRb, and showed with a concentration dependent characteristic (Figure 2D). These results suggested that baicalein promoted G1-phase arrest via inhibiting the transition from G1-phase to S-phase.

### Baicalein induces appotosis of osteosarcoma cells

**Figure 3:**
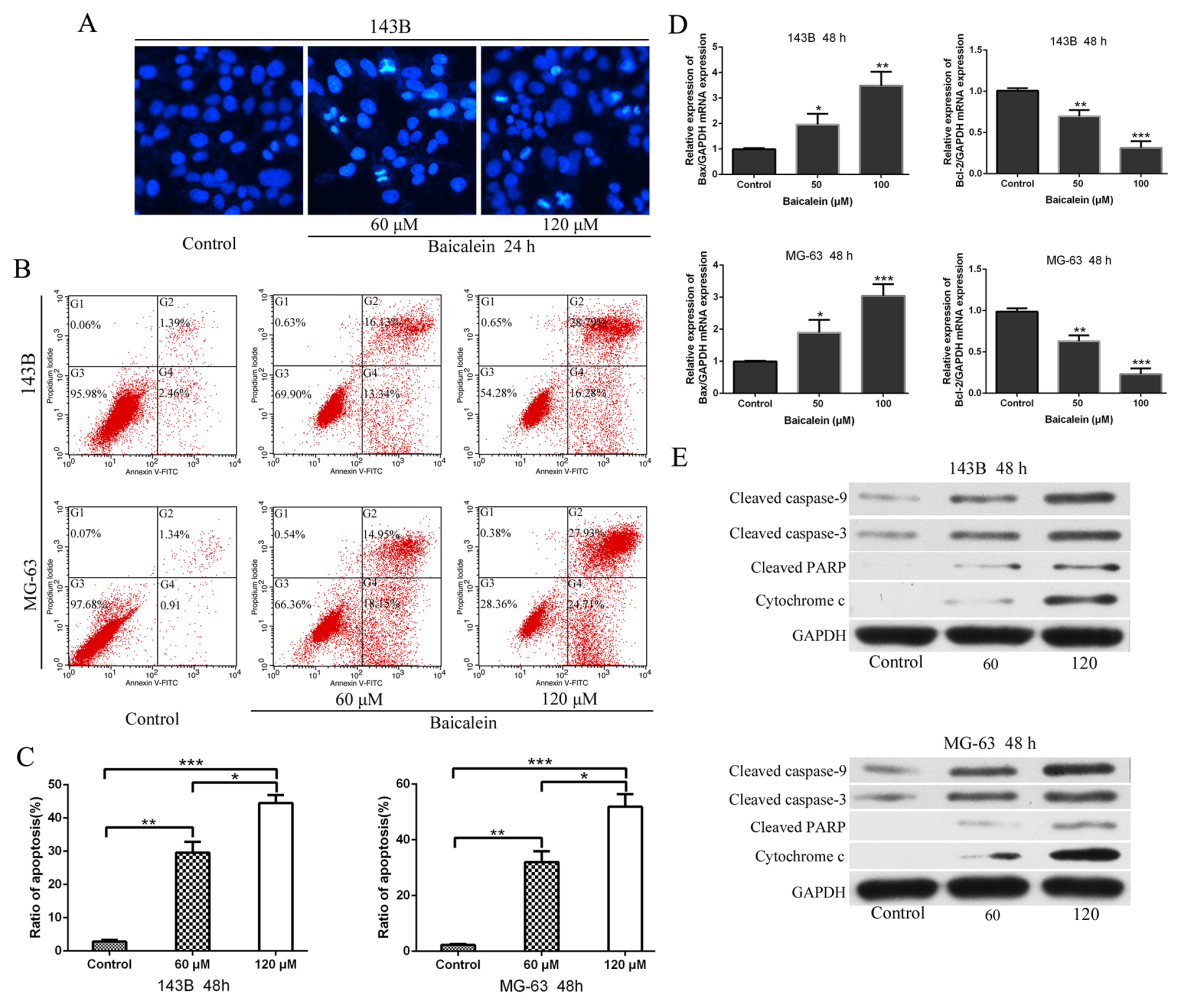
Baicalein induces appotosis of osteosarcoma cells **(A)** 143B cells were incubated with baicalein (60 and 120 μM) for 24 h, and stained with Hoechst 33258. Cell morphology was observed under a fluorescence microscope. Bright-blue fluorescent and condensed nuclei were designated as apoptotic cells (×200). **(B)** Apoptotic cells were detected by flow cytometry using Annexin V-FITC/PI double staining after cells were treated with or without baicalein (60 and 120 μM) for 48 h. **(C)** Quantitative analysis of apoptosis of osteosarcoma cells as shown in B. **(D)** After treatment as in B, total RNA were extracted from cultured osteosarcoma cells and probed with specific primers. Representative results of Bax and Bcl-2 mRNA levels were as determined by RT-qPCR analysis. **(E)** After treatment as in B, proteins were extracted from osteosarcoma cells and probed with appropriate dilutions of specific antibodies. Representative results of cleaved caspase-9, cleaved caspase-3, cleaved PARP, and cytochrome c protein levels were as determined by a Western blot analysis. GAPDH was used as the internal control. Each bar represents means ± SD of three separate experiments, *P < 0.05, **p < 0.01, ***p < 0.001.

Next, we undertook Hoechst 33258 staining assays to ascertain whether baicalein treatment could elicite apoptosis. 60 μm and 120 μm baicalein were used to treat 143B cells (Figure 3A). Hoechst staining exhibited that concentration-dependent induced the condensation and fragmentation of nuclei (a biochemical hallmark of apoptosis). We then examined whether baicalein could induce apoptosis in 143B and MG-63 cells by flow cytometry. The data showed that after treatment with baicalein for 48 h the apoptosis rate increased significantly when compared to the control groups. Moreover, the rate of apoptosis of osteosarcoma cells induced by baicalein was increased in a concentration dependent manner (Figure 3B and 3C). In order to investigate the underlying molecular mechanism of apoptosis induced by baicalein, the expressions of apoptosis-related mRNA and proteins were measured. Therefore, we examined the transcriptional levels of Bax and Bcl-2 mRNA after treatment with baicalein for 48 h, with the increase of concentration of drug, the mRNA level of Bax increased progressively. Conversely, the level of Bcl-2 decreased gradually (Figure 3D). Subsequently, we measured the protein levels of cleaved caspase-9, cleaved caspase-3, cleaved PARP, and cytochrome c. The results exhibited a concentration-dependent manner increase in the expression of apoptosis related proteins (Figure 3E). Thus, we could conclude that baicalein induced apoptosis in 143B and MG-63 cells through mitochondrial-mediated apoptotic related pathway.

### Baicalein reduces the metastatic potential of osteosarcoma cells

**Figure 4:**
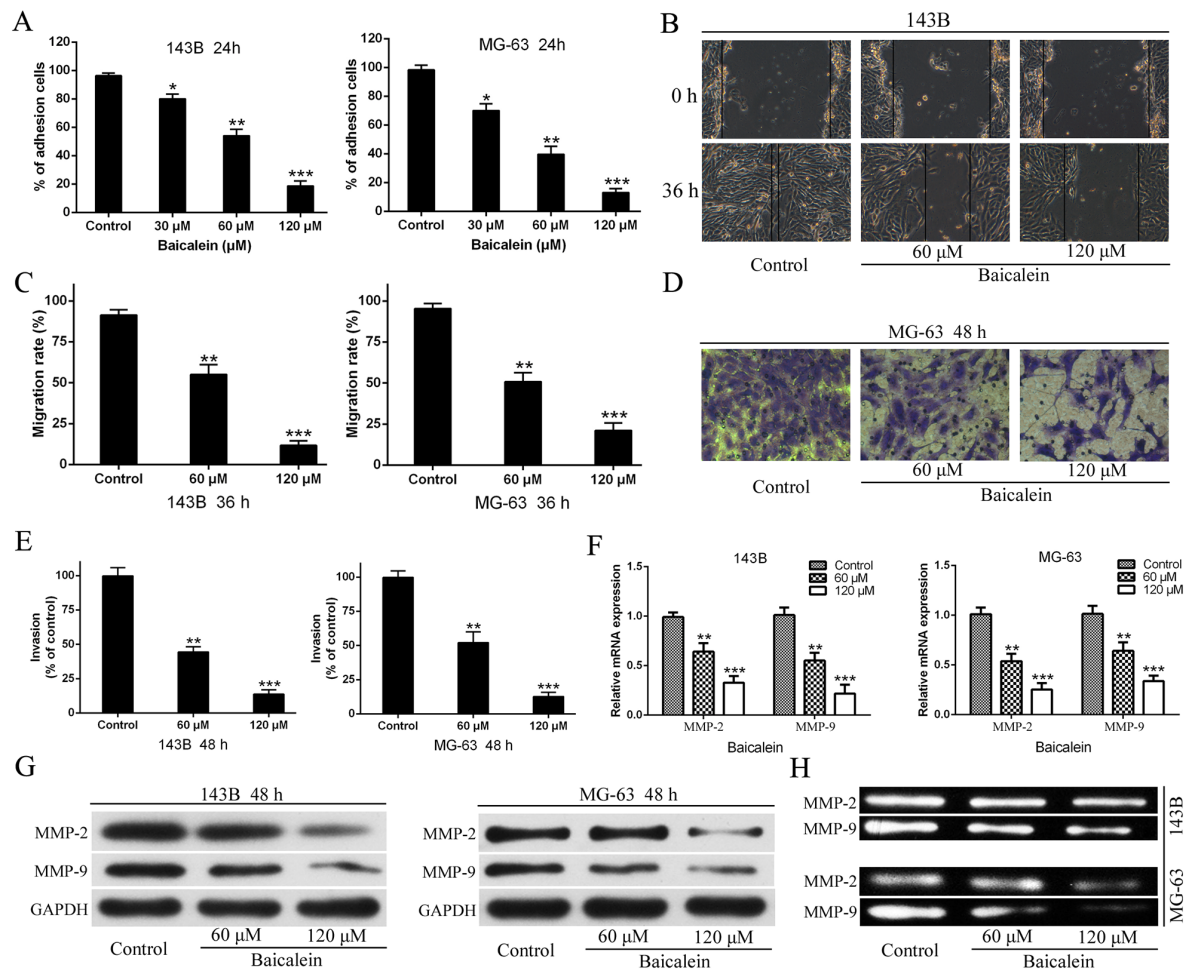
Baicalien inhibits motility potential of osteosarcoma cells **(A)** The CCK-8 assay were carried out to detected adhesion cells of osteosarcoma 143B and MG-63 cells. **(B)** Osteosarcoma cells were incubated with various concentrations of baicalein for 36 h, respectively. Migration to the wounded area was captured (× 100 magnification) and presented. **(C)** The wound area after treatment with baicalein was quantified in three fields for each treatment. **(D)** The invaded MG-63 cells under the membrane were observed under a microscope (× 400) and presented. **(E)** Invasion were quantified by counting the number of 143B and MG-63 cells that invaded into the inner membrane. Untreated cells were used as controls. **(F)** The RT-qPCR were performed to analysis of MMP-2 and MMP-9 mRNA expression following treatment with baicalein in indicated concentration and time. GAPDH was used as an internal control. **(G)** The western blotting analysis of MMP-2 and MMP-9 proteins expression following treatment with baicalein in indicated concentration and time. GAPDH was used as an internal control. **(H)** Gelatin zymography analysis the activities of MMP-2 and MMP-9 of 143B and MG-63 cells after 48 h treatment. Data are expressed as mean ± SD of three independent experiments, *P < 0.05, **p < 0.01, ***p < 0.001 vs. the control group.

Since the abilities of osteosarcoma cells adhesion, migration, and invasion are closely associated with metastatic properties of cancer, three kinds of cytological experiments were performed respectively to assess the effects of baicalein on the metastatic potential of 143B and MG-63 cells. As shown in Figure 4A the number of adhesion osteosarcoma cells significantly decreased in baicalein treated groups compared with the control. This result demonstrated that baicalein could reduce osteosarcoma cells adhesion to the Matrigel™ which was compared to the basement membrane. In order to investigate the effect of baicalein on 143B and MG-63 cell migration, would-healing assay was performed. As shown in Figure 4B and 4C, the potential of cellular migration was inhibited by application of baicalein, the agent conspicuously supressed the migration of osteosarcoma cells in a dose-dependent characteristic. In addition, we further validated the inhibitory effect of baicalein on osteosarcoma cell invasion by Transwell assay. The invasive system uses the Matrigel™ to coat in the upper chamber, which simulates human basement membrane structure. The results verified that baicalein markedly inhibited the invasion of 143B and MG-63 cells and this suppressant also occurred in a concerntration-dependent manner (Figure 4D and 4E).

Degradation of extracellular matrix (ECM) is an important step in tumor invasion and metastasis, and MMPs (matrx metalloproteinases) are known to be crucial for degrading ECM and for facilitating the invasion and metastasis of cancer cells *in vitro* and *in vivo*. Therefore, after 143B and MG-63 cells were treated with different doses of baicalein for 48 h, the expression levels of MMP-9 and MMP-2 were detected and analyzed by using RT-qPCR and Western blot. The results showed that both the mRNA and proteins expression levels of MMP-9 and MMP-2 were markedly lower than those in the control group, and this reduction also exhibited in a concentration-dependent characteristic (Figure 4F and 4G). Further, in order to investigate whether baicalein could lead to a decrease the activities of MMPs, the gelatin zymography assay were performed and the results revealed that the activities of MMP-9 and MMP-2 were reduced in a concentration dependent manner in baicalein-treated cells compared with control group (Figure 4H).

### Baicalein represses the expression of β-catenin and Wnt/β-catenin target genes

**Figure 5:**
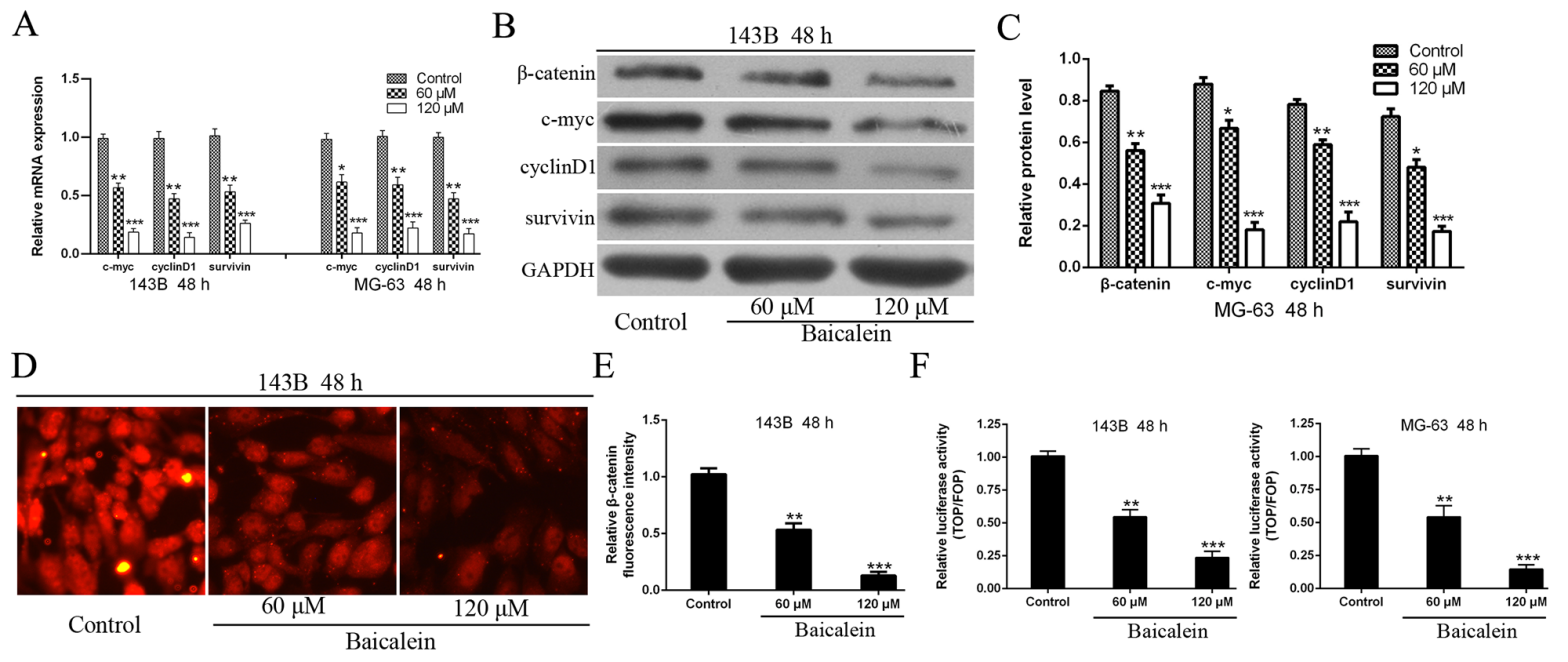
Baicalein represses the expression of β-catenin and Wnt/β-catenin target genes in osteosarcoma cells **(A - C)** RT-qPCR and Western blot analysis the expression of β-catenin and Wnt pathway target genes in osteosarcoma cells following treatment with baicalein in indicated concentration and time. GAPDH was used as an internal control. **(D)** Immunofluorescence assay to detect the expression of β-catenin following treatment with variety concentrations of baicalein. **(E)** Relative quantification of β-catenin protein by fluorescence intensity analysis. **(F)** Osteosarcoma 143B and MG-63 cells were transfected with TOP/FOP flash luciferase reporter vector. Wnt signaling pathway transcriptional activity was attenuated by baicalein and in a dose-dependent manner. Data are shown by means ± SD from three independent experiments. *P < 0.05, **p < 0.01, ***p < 0.001 vs. the control group.

To further explore the molecular mechanism underlying of baicalein induced suppression of osteosarcoma cells progress, Wnt/β-catenin signaling pathway was investigated in-depth. We first detected the downstream target genes of Wnt/β-catenin signaling pathway. As shown in Figure 5A the mRNA expression levels of c-myc, cyclinD1 and survivin were decline significant after treatment with baicalein and accompanied a concentration dependent characteristic in osteosarcoma cells. Then, we have also checked the protein expression levels of β-catenin and downstream target genes (c-myc, cyclinD1, and survivin) of Wnt/β-catenin signaling pathway. The results showed that the protein expression levels of c-myc, cyclinD1, survivin as well as β-catenin were markedly reduced in 143B and MG-63 cells after treatment with baicalein (Figure 5B and 5C). In order to further confirm these results, we conducted immunofluorescence assay to detect β-catenin protein expression. The results showed that application of baicalein led to decrease the fluorescence intensity of β-catenin in a concentration-dependent manner in osteosarcoma 143B cells (Figure 5D and 5E).

The Wnt/β-catenin signal is transmitted into the nucleus via the β-catenin and activates TCF/LEF transcription factors, thereby promoting transcription of related target genes, including c-myc, cyclinD1, survivin and so on [9]. To further verify the inhibitory effect of baicalein on Wnt/β-catenin signaling pathway, we conducted a TOP/FOP-flash luciferase reporter assay to detect transcriptional activity of TCF/LEF transcription factors. As shown in Figure 5F, the activity of TCF/LEF transcription factor, in incremental doses of baicalein treatment group, decreased significantly when compared with the control group and in a concentration-dependent characteristic. It further demonstrated that baicalein could inhibit the activity of TCF/LEF transcription factor and thus blocking the Wnt/β-catenin signaling pathway. All these results confirmed that baicalein could inactivate the Wnt/β-catenin signaling pathway in osteosarcoma cells.

### Upregulation of Wnt/β-catenin signaling pathway relieves the viability, apoptosis and enhanced migration and invasion effects of baicalein in osteosarcoma cells

**Figure 6:**
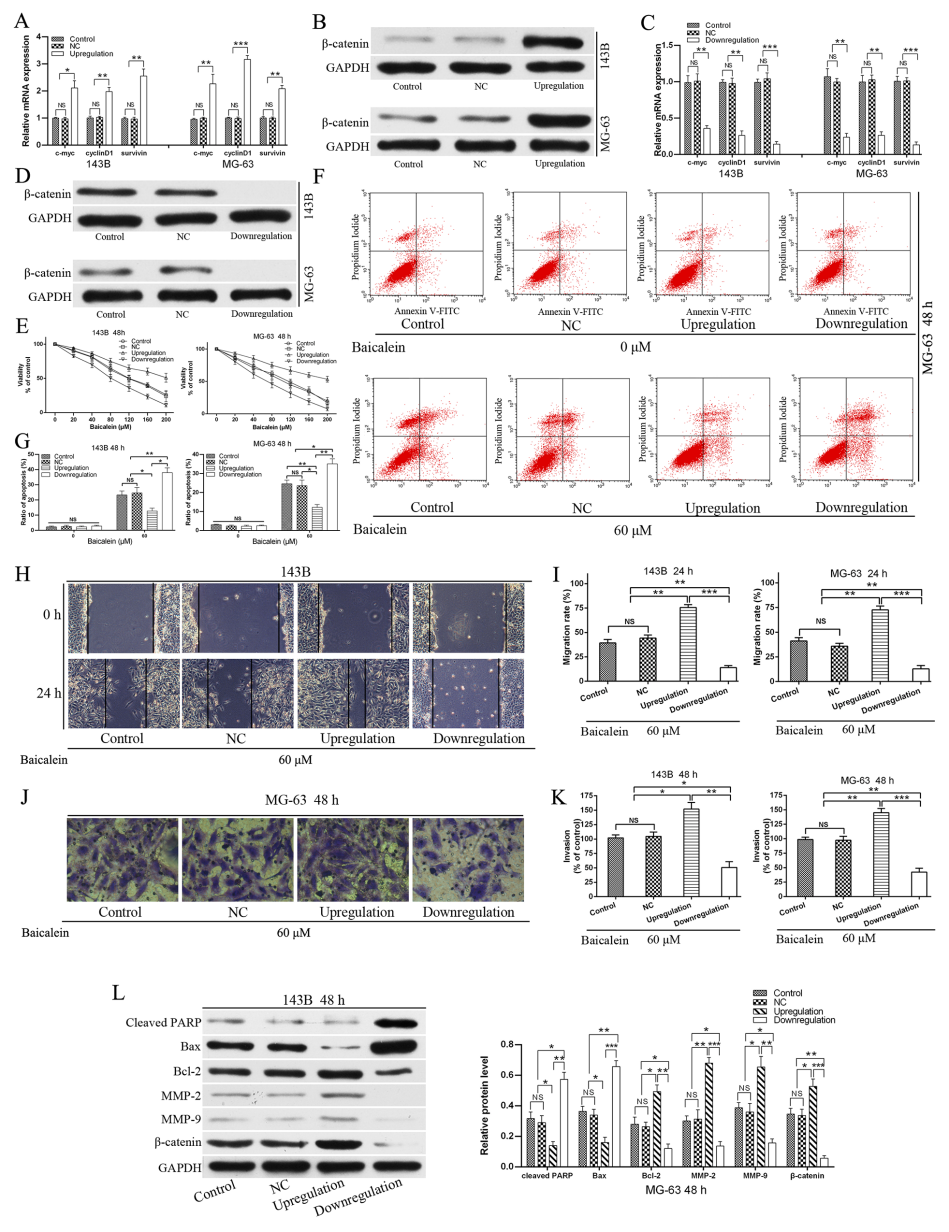
Upregulation of Wnt/β-catenin signaling pathway relieves the viability and apoptosis, and enhanced migration and invasion effects of baicalein in osteosarcoma cells **(A - D)** Osteosarcoma cells were transfected with negative control (NC), β-catenin expressing and β-catenin-shRNA lentivirus respectively. The upregulation, downregulation and NC of Wnt/β-catenin signaling pathway in osteosarcoma 143B and MG-63 cells were confirmed by RT-qPCR and wertern blot respectively. **(E)** Stably transfected osteosarcoma cells and non-transfected cell (Control) were treated with increasing doses of baicalein (20–200 μM) for 48 h. CCK-8 assay showed that upregulation of Wnt/β-catenin signaling pathway (β-catenin overexpressing) significantly blocked the inhibition of proliferation of 143B and MG-63 cells by application of baicalein. Downregulation of Wnt/β-catenin signaling pathway (β-catenin-shRNA) significantly enhanced the anti-proliferation effect of 143B and MG-63 cells by application of baicalein. **(F)** Flow cytometry analysis of apoptotic ratio of upregulation and downregulation of Wnt/β-catenin signaling pathway in MG-63 cells which were treated with or without baicalein for 48 h. **(G)** Quantitative analysis of apoptosis of osteosarcoma cells in 143B and MG-63 cells. **(H)** The osteosarcoma 143B cells migration was estimated by wound healing assay. Stably transfected osteosarcoma cells and non-transfected (control) cells were treated with or without 60 μM baicalein for 24 h. Migration to the wounded area was captured (× 100 magnification) and presented. **(I)** The wound area after treatment with baicalein was quantified in three fields for each treatment in 143B and MG-63 cells. **(J)** Stably transfected osteosarcoma cells and non-transfected (control) cells were treated with or without 60 μM baicalein for 48 h. The invaded MG-63 cells under the membrane were observed under a microscope (× 400) and photographed. **(K)** Invasion were quantified by counting the number of 143B and MG-63 cells that invaded into the inner membrane. **(L)** Upregulation of Wnt/β-catenin signaling pathway prevented the expression of cleaved PARP, inhibited the levels of MMP2 and MMP9 and increased the ratio of Bcl-2/Bax in response to baicalein (60 μM). Downregulation of Wnt/β-catenin signaling pathway of 143B and MG-63 coincided with the opposite results. There was no difference between the NC group and the control group. The protein levels of cleaved PARP, Bax, Bcl2, MMP-2, MMP-9, and β-catenin were determined by Western blot, GAPDH was used as an internal control. NC, negative control. Data are shown by means ± SD from three independent experiments. *P < 0.05, **p < 0.01, ***p < 0.001.

In view of the fact that the Wnt/β-catenin signaling pathway plays a key role in cell growth, survival, differentiation, stem cell maintenance, metastasis, and tumor formation [9] and baicalein represses the expression of β-catenin and Wnt/β-catenin target genes. Thus, we hypothesized that baicalein exhibited the anti-proliferation and induction apoptosis and decrease motility effects may partly through down-regulating the Wnt/β-catenin signaling pathway. In order to prove the hypothesis, we employed recombinant lentivirus to construct the negative control (NC), upregulation and downregulation of Wnt/β-catenin signaling pathway of osteosarcoma cells. All these transfected osteosarcoma cells were confirmed by both RT-qPCR and western blot (Figure 6A - 6D). After obtaining stable transfected osteosarcoma cell lines of 143B and MG-63, we performed a number of functional experiments. As Figure 6E shown that exogenous expression of β-catenin, which upregulation Wnt/β-catenin signaling could weaken the anti-proliferative effect of baicalein in 143B and MG-63 cells, and conversely, enhanced anti-proliferative effects were observed in β-catenin-shRNA transfected cells. And transfection of the NC lentivirus did not increase or impair the anti-proliferation effect of baicalein compared with control after treatment with baicalein for 48 h. Therefore, we could conclude that baicalein exhibits the anti-proliferation effect partly through down-regulating the Wnt/β-catenin signaling pathway.

Furthermore, we verified the correlation between baicalein induced-apoptosis and inactivation of Wnt/β-catenin signaling pathway. Exogenous expression of β-catenin could reverse the apoptosis rate of baicalein treated osteosarcoma cells. In contrast, downregulation of Wnt/β-catenin signaling pathway significantly increase the induced-apoptosis effect of baicalein compared with the control and upregulation group. Transfection of the NC lentivirus did not increase or impair the induced-apoptosis effect of baicalein compared with the control group (Figure 6F and 6G). Therefore, we could conclude that baicalein exhibits the induction apoptosis effect partly through down-regulating the Wnt/β-catenin signaling pathway.

In addition, the inhibition of migration (Figure 6H and 6I) and invasion (Figure 6J and 6K) capability of osteosarcoma cells by baicalein were markedly reversed by the exogenous expression of β-catenin, and downregulation of Wnt/β-catenin signaling pathway significantly enhanced the inhibitory effect of baicalein in migration and invasion of 143B and MG-63 cells (Figure 6H - K). On the one hand, upregulation the Wnt/β-catenin signaling pathway remarkably suppressed baicalein induced activation of PARP and increased the ratio of Bcl-2/Bax (Figure 6L). On the other hand, the exogenous expression of β-catenin also led to block of baicalein induced inhibition of MMP-9 and MMP-2 (Figure 6L). Thus, all these results demonstrated that the Wnt/β-catenin signaling pathway might participate in baicalein induced anti-proliferation and anti-metastasis in osteosarcoma cells. Upregulation of Wnt/β-catenin signaling pathway relieved the viability, apoptosis and enhanced migration and invasion effects of baicalein in osteosarcoma cells.

### Baicalein inhibits osteosarcoma cell growth *in vivo*

**Figure 7:**
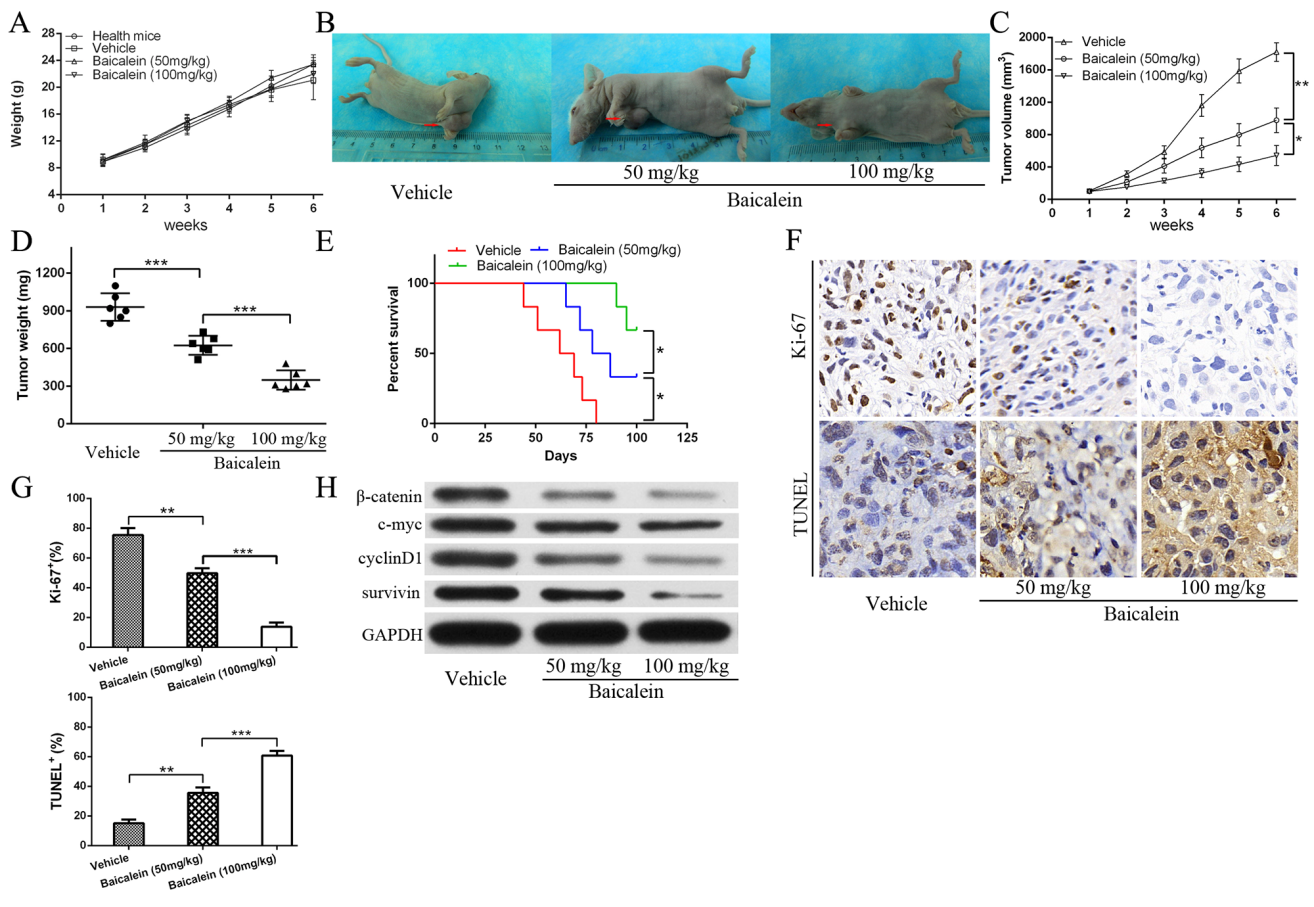
Baicalein inhibits osteosarcoma143B cell growth *in vivo* **(A)** The mice body weight (in grams, recorded every week) were recorded and compared. **(B)** Representative images of 143B xenograft vehicle and treatment with low and high dose of baicalein. **(C)** The tumor volume were recorded and compared (volume in mm^3^, recorded every week). **(D)** Tumor weight was obtained at the end of the experiment. **(E)** Survival rate of mice after the indicated treatment were presented. **(F)** Immunohistochemical (IHC) analysis of Ki-67 and TUNEL staining for tumors of all treatment groups ( × 400, magnification). **(G)** Quantitative analysis IHC and apoptotic cells as shown in F, each group were counted on the basis of viewing eight random fields in each glide. **(H)** The proteins expression of β-catenin, c-myc, cyclinD1, and survivin in xenograft tumor tissues of all treatment groups. GAPDH was used as a loading control. all data are expressed as the mean ± SD of three independent experiments. NS, not significant. *P<0.05, **P<0.01.

At last, we examined whether baicalein could restrain osteosarcoma cells growth and survial *in vivo*. We used an established mouse xenograft model bearing 143B cells as previously described [26]. As shown in Figure 7A, after treatment with or without low and high doses baicalein, the body weight in all groups showed no statistically significant difference when compared with the healthy mice. However, baicalein markedly inhibited the 143B xenograft growth in nude mice in a dose-dependent characteristic (Figure 7B - 7D). The data indicated that baicalein inhibited osteosarcoma cells growth without significant systemic toxicity. Furthermore, baicalein administration improved mice survival as shown in Figure 7E, especially in the high-dose group. In order to further investigate the mechanism, we examined the proliferation and apoptosis related indicators by immunohistochemistry and TUNEL assay. As shown in Figure 7F and 7G, baicalein treatment led to decrease the proliferation level and increase apoptosis expression as measured by Ki-67 staining and TUNEL staining of the subcutaneous tumor tissue sections, which verified that baicalein could suppress the growth of tumor and induced obviously cell apoptosis in the tumor mass. What is more, tumor metastasis foci were not found in the extracted organs of high-dose baicalein treatment group according to the HE staining (no figure). Finally, we examined whether baicalein could repress the Wnt/β-catenin signaling pathway *in vivo*. The results showed a reduction of β-catenin, c-myc, cyclinD1 and survivin protein levels after baicalein treatment (Figure 7H), which were consistent with the data *in vitro*.

## DISCUSSION

Osteosarcoma is one of the malignant that mainly occurs in children and adolescents [1]. With the development of diagnosis and treatment of osteosarcoma, the five-year survival rate for osteosarcoma patients improved a lot in these years, but still undesirable [4]. The drugs used for osteosarcoma patients are mainly the same as that used in 1980s [27], such as cisplatin and doxorubicin, which have side effects and may be life-threatening for many osteosarcoma patients who cannot tolerate [2]. Therefore, it is essential to explore more safer and efficient drugs targeting malignant cells.

Baicalein is a widely used Chinese herbal medicine that is extracted from Huang Qin, which comes from the root of *Scutellaria baicalensis Georgi* and has multiple pharmacological activities [20, 21]. In recent decades, numerous studies have confirmed that baicalein is capable of suppressing tumor cells survival and growth, inducing apoptosis and cycle arrest of cells in human gastric cancer, gallbladder cancer, hepatocellular carcinoma, pancreatic cancer, colon cancer, lung cancer, prostate cancer, breast cancer, and hematological malignancies cell lines [20, 21, 28-37]. Mu *et al.* [28] demonstrated that 15, 30, 60, 120, 20μM baicalein could exhibit potent inhibitory effects on gastric cancer cell survival and growth and colony formation. On the one hand, it may be because of baicalein induced cell cycle arrest; on the other hand, it may be due to baicalein induce apoptosis of gastric cancer cells through the mitochondrial-mediated pathway. Baicalein also exhibited excellent tumor inhibitory effects in a *in vivo* subcutaneous xenograft animal model. Zhang *et al.* [38] reported that baicalein could inhibit CDK1 via oxidizing CDC25C, leading to suppress proliferation in cancer cells. Further, baicalein could activate the intrinsic apoptotic pathways by oxidizing caspases and extrinsic death receptor pathway bypassing, therefore inducing apoptosis in cancer cells specifically rather than in normal cells and activating lymphocytes. Zhang and his colleagues [38] also discovered that BA-j, as a derivative of baicalein and selectively inhibited CDK1, could induce apoptosis in cancer cells via regulating reactive oxygen species. Although detailed studies have been carried out in some other tumors, the study of baicalein in osteosarcoma is still not sufficient and deep enough. In this study, we tested the antitumor effects of baicalein in 143B and MG-63 cell lines, two widely used osteosarcoma cell lines. The underlying molecular mechanisms of baicalein in suppressing osteosarcoma cells growth and survival *in vitro* and *in vivo* were also been investigated. To the best of our knowledge, this work is the first study of the function and mechanism of action of baicalein in osteosarcoma.

Firstly, we evaluated the anti-poliferation effect of baicalein on osteosarcoma cells using a CCK-8 assay and a colony formation assay respectively (Figure 1). The results showed that baicalein can inhibit cell growth and survival in a concentration- as well as time-dependent manner. These two experiments effectively demonstrate that baicalein can not only inhibit the proliferation of osteosarcoma cells in a short time, but also effectively prevent the growth of single cell over a relatively long period of time.

The effect of baicalein on cell cycle distribution in osteosarcoma cells were also evaluated. Flow cytometry combined with PI staining to detected the DNA content of each cell phase. It was found that baicalein could induce G1-phase arrest in osteosarcoma cells, and this blocking was in a concentration-dependent manner (Figure 2). Cell cycle regulation is mainly through the synthesis and degradation of cyclin in different cell phases, which depend on their positive regulatory subunits named as cyclin dependent kinase (CDK), and negatively regulate by CDK inhibitor (CDI) [39]. Since the phenomenon of cell cycle has been recognized, a lot of studies and literatures have demonstrated the correlation of cell cycle distribution deregulation in variety of human cancers including osteosarcoma [25]. It has been reported that baicalein could induce cell cycle arrest at different phase, which depended upon the type of tumor cells and the mechanism of action [20, 40]. However, there are relatively few studies about the effect of baicalein on the cell cycle of osteosarcoma. In the present study, baicatelin was found to regulate the cell cycle distribution of osteosarcoma. Specifically, as the concentration of baicalein increased, the percentage of 143B and MG-63 in the G1 phase increased gradually, showing the cell cycle arrest effect; at the same time, the proportion of osteosarcoma cells in S phase and G2/M phase decreased, but the reduce was in a disorderly manner.

To investigate the underlying molecular mechanism of baicalein on cell cycle regulation, we examined the expression levels of Cyclin D1, Cyclin E1, CDK4, CDK6, c-myc, pRb, p21 and p27 in osteosarcoma cells, which treated with different doses of baicalein. RT-qPCR and western blot analysis revealed that Cyclin D1, Cyclin E1, CDK4, CDK6, c-myc, and pRb were downregulated, but p21 and p27 were upregulated. It has been reported that Cyclin D1, Cyclin E1, pRb, c-myc, and SKP2 could facilitate G1-S phase progression [41]. At present, it is believed that the expression of p21 and p27 could directly lead to G1 phase arrest. p21 and p27 combined with cyclin E/CDK2 complex and weaken the activity of CDK2 by inhibiting phosphorylation of CDK2, then resulted in restriction on G1-S transition [42]. In this study, we demonstrated that baicalein could decrease Cyclin D1, Cyclin E1, CDK4, CDK6, c-myc and pRb expression in a concentration-dependent characteristic, but the protein expression levels of p21 and p27 increased in a concerntration-dependent manner (Figure 2). These results might partly explain the effects of baicalein on G1 phase arrest. Thus, baicalein treatment significantly inhibited 143B and MG-63 cell proliferation, which was partially related to cell cycle arrest.

Since apoptosis is considered as a very important mechanism to inhibit survival of cancer, so Hoechst 33258 staining assay and Annexin V-FITC/PI double staining assay were performed to detection apoptosis in osteosarcoma cells that were induced by baicalein. Flow cytometric analysis exhibited that baicalein produced a concentration-dependent increase in the percentage of Annexin V-staining apoptotic cell population, showing the sequential events, that is, apoptosis occurs after cell cycle arrest. Many morphological changes of cells indicate the occurrence of apoptosis, which include cell shrinkage, membrane blebbing, fragmentation and condensation of nuclei, and the formation of apoptotic bodies. Hoechst 33258 staining clearly revealed bright-blue fluorescent and condensed, fragmented nuclei, which was a biochemical hallmark of apoptosis cells [43, 44] (Figure 3). There results further comfirmed that baicalein could induce osteosarcoma cells apoptosis. Drug stimulation could cause caspase cascade, which ultimately leads to the activation of the caspase responsible for apoptosis. In general, there are two major apoptotic pathways: the extrinsic (death receptor-mediated) pathway and the intrinsic (mitochondrial-mediated) pathway, both pathways contribute to the drug induced apoptosis of tumor cells [44, 45].

In order to investigate the molecular mechanism of baicalein-induced apoptosis, the apoptosis related proteins and mRNA were detected. The immunoblotting and RT-qPCR showed that the expression levels of cleaved caspase-9, cleaved caspase-3, cleavage of PARP, cytochrome c and Bax increased and Bcl-2 decreased gradually according to the dose of baicalein. Number of these genes and proteins are related to the intrinsic mitochondrial-mediated apoptotic pathway. Members of Bcl-2 protein family either prevent apoptosis or promote apoptosis [46]. Bax could promote apoptosis effect by binding to Bcl-2 protein and antagonizing its action [45, 46]. In this work, we exhibited that baicalein up-regulated the expression level of Bax and down-regulated the level of Bcl-2 mRNA, which increased the permeability of mitochondrial membrane leading to release of cytochrome c and then activating caspase-9 and caspase-3/7 subsequently [45], shearing substrate PARP, eventually induce cell apoptosis (Figure 3). From these results, we could conclude that baicalein induced osteosarcoma cells apoptosis through activating the intrinsic mitochondrial-mediated apoptosis pathway.

In addition to cell proliferation, invasion and metastasis are considered to be two important biological characteristics of malignant tumors. Cell adhesion, migration, and invasion are considered to play critical roles in tumor metastases [47]. In order to elucidate the effect of baicalein on cell motility, we performed adhesion, scratch and transwell assay respectively to determine the capability of cell adhesion, migration and invasiveness after treatment with baicalein. From these results, we found that baicalein could inhibit motility of 143B and MG-63 cells in a concentration-dependent manner (Figure 4). First of all, tumor cells must detach from their primary lesion and interact with the ECM and the basement membrane at each stage in the metastatic cascade. Then, the degradation of ECM and the basement membrane, isolation of cells and connective tissues, invasion adjacent tissues and vessels, seem to be the important starting steps [19]. The MMPs have verified to be involved in degradation of most ECM and believed to be associated with invasiveness, metastasis and angiogenesis. It has been reported that the depth of invasion, the distant of metastasis, and the permeation of vessels are positively correlated with the expression levels of MMP-2 and MMP-9 [19, 48]. And MMP-2 and MMP-9, two of the major proteases, which contribute to migration and invasion in osteosarcoma pathogenesis [19, 48]. In this work, we showed that baicalein could not only reduce the protein expression but also decrease the enzymatic activity of MMP-2 and MMP-9, this results could partially explain the decline in osteosarcoma motility after treatment with baicalein. In animal models, we also found that there were no metastatic tumors of osteosarcoma from the extracted organs of the nude mice in the high-dose baicalein group according to the HE staining. Taken together, our results suggested that baicalein could inhibit the potential of osteosarcoma cell metastasis both *in vitro* and *in vivo*.

The Wnt/β-catenin signaling pathway has been demonstrated to play a critical role in modulating diverse processes, including cell proliferation, survival, differentiation, metastasis and polarity, specification of cell fate, and self-renewal in stem cells, and maintain stem cell properties [9, 49]. Abnormal activation of Wnt/β-catenin signaling pathway has been discovered in variety of tumors [9, 50]. Watson *et al.*[51] reported that canonical Wnt/β-catenin signaling was a novel genetic driver of Schwann cell tumor development and progression, and down-regulation of this pathway was sufficient to reduce the tumorigenic phenotype of human malignant peripheral nerve sheath tumors. So, inhibitors of Wnt/β-catenin signaling pathway alone, or plus with RAD-001 (mTOR inhibitor), represents a novel and effective target for therapeutic intervention in Schwann cell tumors. Radulescu *et al.* [52] demonstrated that Wnt/β-catenin signaling pathway activation can act as an initiator in gastric neoplasia. Both deletion of GSK3 α and β or APC, which lead to immediate nuclear β-catenin accumulation and Wnt/β-catenin signaling pathway gene expression. Intestinal-type adenomas and FGPs (fundic gland polyps) were the results of the Wnt/β-catenin signaling pathway activation. Wnt/β-catenin signaling pathway has been also closely associated with the occurrence and development of osteosarcoma [8, 15]. Some scholars have reported that the ligands, receptors and downstream target genes were highly expressed in osteosarcoma cells [11, 13, 53, 54]. It is believed that the activation of the Wnt/β-catenin signaling pathway is vital and common in osteosarcoma genesis [13]. In addition, it appears that blocking Wnt/β-catenin signaling pathway could suppress tumorigenesis and metastatic potential in osteosarcoma [8]. And more and more drugs that suppressed the Wnt/β-catenin pathway have been proved to be efficient in osteosarcoma treatment [55–59]. Therefore, the inhibition of Wnt/β-catenin signaling pathway may have important therapeutic applications for preventing and treating osteosarcoma. In our study, we demonstrated and verified for the first time that baicalein downregulated the Wnt/β-catenin pathway and its downstream target genes, including cyclinD1, c-myc and survivin (Figure 5). Which lead to osteosarcoma cell growth inhibition, cell cycle arrest, induction apoptosis, and metastasis restriction. Other scholars have also confirmed that baicalein could inhibit growth and metastasis of solid tumors and hematologic malignancies via inactivation Wnt/β-catenin pathway [23, 60]. Due to the central role of Wnt/β-catenin pathway in osteosarcoma genesis, development and progression, we hypothesized that this pathway is a target for baicalein. In order to prove the hypothesis, we employed recombinant lentivirus to construct the negative control (NC), upregulation and downregulation of Wnt/β-catenin signaling pathway of osteosarcoma cells. And most importantly, Wnt/β-catenin signaling pathway activation by overexpression of β-catenin not only significantly reversed the anti-proliferation and induction apoptosis of baicalein, but also attenuated the baicalein-induced motility restriction, indeed promoting its motile ability. In short, upregulation of Wnt/β-catenin signaling pathway relieved the viability, apoptosis and enhanced migration and invasion effects of baicalein in osteosarcoma cells. On the other hand, we also exhibited that, together with baicalein treatment, Wnt/β-catenin signaling pathway inhibition by β-catenin-shRNA could further reduce cell viability, promote apoptosis, inhibit migration and invasion of osteosarcoma cells compared with the NC group and control. These data further support the critical role of Wnt/β-catenin pathway in baicalein-mediated inhibition effects in osteosarcoma. Thus, all these results further demonstrated that baicalein-mediated anti-proliferation amd anti-metastasis of osteosarcoma cell may be associated mechanistically with the inactivation of Wnt/β-catenin pathway (Figure 6).

In human xenograft tumor models in BALB/c nude mice, baicalein was shown to suppress tumor growth markedly without affecting its body weight. High dose of baicalein significantly prolonged the survival rate of nude mice. The therapeutic effect of baicalein was confirmed to be mediated in part via inhibiting proliferation and induction apoptosis detected by Ki-67 and the TUNEL staining of tumor sections. After baicalein treatment, the expression of β-catenin and target gene proteins (cyclinD1, c-myc, survivin) in tumor tissue decreased significantly, indicating that the potential molecular mechanism might be through inhibiting the Wnt/β-catenin signaling pathway (Figure 7).

**Figure 8:**
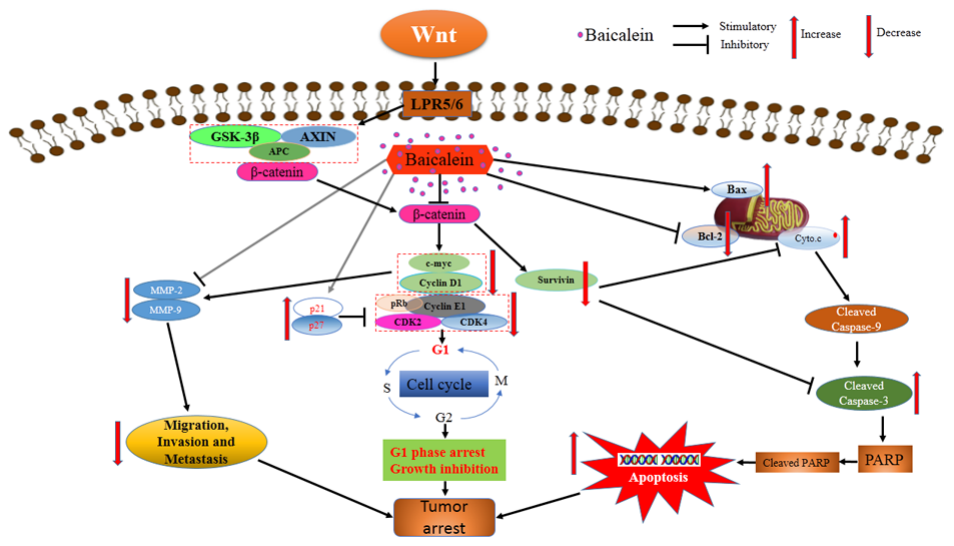
Schematic view depicting mechanisms of baicalein inhibits progression of osteosarcoma cells through inactivation of the Wnt/β-catenin signaling pathway

Taken together, we found that baicalein could inhibit osteosarcoma cells proliferation, regulate the expression of Bcl-2 family members, induce apoptosis by caspase activation and then suppressing survival. Baicalein reduced the expression of c-myc, cyclinD1 and increased p21, p27, which contributed to G1-phase arrest of osteosarcoma cells. In addition, we showed that baicalein suppressed osteosarcoma cells adhesion, migration, and invasion by decreasing the expression and activity of MMP-2 and MMP-9. The multiple-functions of baicalein led to the dramatically inhibitory effect on osteosarcoma cells *in vitro* and inhibited osteosarcoma growth and survival *in vivo*. Baicalein repressed the expression of β-catenin and Wnt/β-catenin signaling pathway target genes, however upregulation of Wnt/β-catenin signaling pathway could relieve the viability, apoptosis and enhance migration and invasion effects of baicalein in osteosarcoma cells. Thus the underlying molecular mechanism for baicalein exerting anti-proliferation and anti-metastatic effect on osteosarcoma cells may be due to inactivation of Wnt/β-catenin signaling pathway (Figure 8). Although further in-depth studies are needed to ascertain the therapeutic target of the Wnt/β-catenin signaling pathway in baicalein-induced cell survival and metastatic inhibition, the results of our study provide valuable evidences from cells and animals experiments to support baicalein, a traditional Chinese herbal medicine, as a novel and effective candidate agent for the chemoprevention and/or treatment of osteosarcoma.

## MATERIALS AND METHODS

### Chemicals and reagents

Baicalein, Hoechst 33258, dimethyl sulfoxide (DMSO) were purchased from Sigma-Aldrich (St. Louis, MO, USA). Baicalein was dissolved in 100 % DMSO to prepare a 200 mM stock solution, which was stored at −20 °C. Cell Counting Kit-8 (CCK-8) was purchased from Dojindo (Japan). Primary antibodies against human Bax, Bcl-2, p21, p27, cyclinD1, CDK2, pRb, MMP-2, MMP-9, β-catenin, c-myc, survivin were purchased from Abcam (Cambridge, UK). RIPA Lysis Buffer, primary antibodies against cleaved caspase-9, cleaved caspase-3, cleaved PARP, cytochrome c, GAPDH and horseradish peroxidase (HRP)-labeled secondary antibody were obtained from Cell Signaling Technology (CST, Beverly, MA, USA). Amersham ECL Western blotting detection reagents and analysis system were purchased from GE Healthcare (Buckinghampshire, UK).

### Cell culture

The human osteoblast cell line hFOB1.19 and osteosarcoma cell line MG-63 were obtained from the Cell Bank of the Chinese Academy of Sciences (Shanghai, China) and 143B was purchased from China Centre for Type Culture Collection (CCTCC, Wuhan, China). MG-63 cells was cultured in DMEM (Hyclone, Utah, USA) containing 10% fetal bovine serum (FBS, Gibco, Australia) and 1% antibiotics (penicillin 100 U/ml, streptomycin 100 μg/ml, Genom, Hangzhou, China). Cells were propagated in a humidified environment at 37°C with 5% CO_2_ and 100% humidity. 143B cells were maintained in the same conditions, except that α-MEM/EBSS (Hyclone, Utah, USA) medium. Human osteoblast cell line hFOB1.19 was maintained in DMEM/F-12 (Hyclone, Utah, USA) supplement 10% FBS (FBS, Gibco, Australia) and geneticin (400 μg/ml, Sigma) at 34°C in a saturated humidified with 5% CO_2_ atmosphere incubator. Cell viability was determined using trypan blue staining. Culture medium was replaced every three days.

### Generation of stably transfected cell lines

The negative control (NC), β-catenin overexpression and β-catenin-shRNA lentivirus were purchased from Shanghai GeneChem Co., Ltd (Shanghai, China). To generate stably transfected cell lines, osteosarcoma cells were infected with the viral supernatant according to the manufacturer’s protocol. Transfected cells were incubated for 48 to 72 h prior to selection of stable transfectants by addition of puromycin (5μg/ml) for two weeks in selective medium, then those stably transfected cell lines can be obtained.

### Cell viability assay

Briefly, cells were seeded at a final concentration of 5 × 10^3^ cells/well in 96-well plates. After attachment for 24 h, the medium was changed for fresh medium containing 0, 20, 40, 80, 120, 160, and 200 μM baicalein and cells were incubated for another 24, 48 or 72 h. Subsequently, the viability of cells was measured using the CCK-8 assay at indicated time points. Before testing, CCK-8 solution (10 μl) was added to each well containing 100 μl mixture of culture medium. The plates were incubated for 2 h at 37°C in the incubator. Cell viability were counted by absorbance measurements at 450 nm using auto microplate reader (Tecan Sunrise, Austria). The OD450 value was proportional to the viability of cell. All experiments were performed in triplicate.

### Colony-formation assay

Osteosarcoma 143B and MG-63 cell lines were plated in 6-well plates at a density of 1 × 10^2^ cells per well and treated with or without baicalein for 24 h. Cells were incubated for another two weeks in baicalein-free conditions. Colonies were fixed with methanol and stained with Wright-Giemsa solution. Plates were photographed and the number of colonies was calculated.

### Flow cytometry

For cell cycle analysis, cells were harvested and then fixed in 70% ethanol, treated with 300 μg/ml RNase A (Sigma-Aldrich, St. Louis, MO, USA), and their nuclei were stained with 10 μg/ml propidium iodide (PI) (Sigma-Aldrich, St. Louis, MO, USA). The stained nuclei were measured by FACSCalibur (Becton-Dickinson, San Jose, CA, USA). The data was analysed using Modfit software. The apoptosis assay was performed with an Annexin V-FITC (fluoresceine isothiocyanate) /PI apoptosis detection kit (BD, USA) according to the manufacturer’s protocol. Cells were washed twice with cold PBS buffer and then resuspended in 1×binding buffer at a concentration of ∼1×10^6^ cells/ml, and 5 μl of Annexin V-FITC conjugate and 5 μl of PI solution were added to each 500 μl cell suspension. Cells were stained by Annexin V-FITC/PI for 0 min at room temperature, protected from light. Stained samples were analyzed using FACSCalibur and the apoptosis rate was determined using Flowjo software.

### Hoechst 33258 staining for apoptosis

Hoechst 33258 Staining Kit (Sigma-Aldrich, St. Louis, MO, USA) was used for this assay. The cells were incubated in a 6-well plate for 24 h and further treated with or without baicalein for 24 h. Subsequently, cells were stained according to the instructions and then observed under an inverted fluorescence microscope (Olympus) (×200). Cells were designated as apoptotic cells based on nuclear morphology changes, such as bright-blue fluorescent and condensed nuclei.

### Adhesion assay

Osteosarcoma cells were pretreated with various doses of baicalein (30, 60, 120 μM) for 24 h, and single-cell suspensions were harvested then plated and cultured for 2 h on 96-well microplates that had been pre-coated with 100 μg/ml Matrigel™ (Sigma-Aldrich, St Louis, MO, USA). Subsequently, each group was washed for 30 - 60 min to remove the non-adherent cells. Then, the CCK-8 assay was performed and cell adhesion capability were counted by absorbance measurements at 450 nm using auto microplate reader.

### Wound healing assay

The wound healing assay is a widely used method for judging the ability of cell migration. 143B and MG-63 cells were cultured in 12-well and allowed to reach confluence. Scratch was made using a 1-ml sterile pipette tip across the cell monolayer. Media was removed and rinsed with PBS twice and treated with varying concentrations of baicalein in α-MEM/EBSS or DMEM without FBS. Would closure was measured after 36 h treatment under a microscope and images were captured accordingly. The migration rate was calculated following the formula: migration rate (%) = (average original width - average final width) / average original width × 100%. All experiments were performed in triplicate.

### *In vitro* cell invasion assay

For the cell invasion assays, the Transwell membranes (Corning Inc., New York, NY, USA) were purchased. Transwell invasion assays were performed according to the manufacturer’s protocol. Briefly, 1×10^5^ cells without FBS medium were seeded in the upper chamber of the Transwell invasion system with Matrigel™. The low chambers were filled with 600 μL culture medium containing 10% FBS as chemoattractant. Then, the Transwell invasion system was placed into cell culture incubator for 48 hours. From the upper chamber, cells on the upper surface of the membrane were removed. Finally, cells that invaded the lower chamber were stained with 0.2% crystal violet (Sigma-Aldrich, St Louis, MO, USA) for 20 min. Quantitated by counting in five different fields under microscopy (×100) and image were captured.

### Gelatin zymography assay

The activities of MMP-2 and MMP-9 were assayed by gelatin zymography. Briefly, according to the manufacturer’s instructions, cells were cultured in indication condition for 48 h, and the conditional medium were collected. After electrophoresis, the gels were incubated at 37°C for 48 h in activation buffer (50 mM Tris-HCl, pH 7.5, 5 mM CaCl_2_, pH 8.0, μM ZnCl_2_, 0.02% NaN_3_). Then, the gels were stained with 0.05% Coomassie blue (R-250) (Bio-Rad, Hercules CA, USA) for 3 h and destaining in methyl alcohol, acetic acid destaining solution until clear bands were visible, then images were captured.

### Reverse transcription quantitative PCR (RT-qPCR)

Total RNA was extracted from cell lines using TRIzol reagent (Invitrogen, Carlsbad, CA, USA) according to the manufacturer’s instructions. The RNA quantity and integrity were examined by ND-1000 spectrophotometer. cDNA was synthesized from total RNA using Transcriptor First Strand cDNA Synthesis Kit (Roche). Quantitative PCR reactions were set up in triplicate and performed on a 7900 PCR machine using SYBR Green PCR Master Mix (Takara, Kyoto, Japan) according to the manufacturer’s protocol. Gene expression levels were calculated via the 2^-ΔΔCt^ method and normalised to GAPDH expression. The primer sequences used are listed in Table 2.

**Table 2:** Primer sequences used for real-time PCR analysis

Genes	Primer sequence	
Cyclin D1	Forward Reverse	5’-ACAAACAGATCATCCGCAAACAC-3’ 5’-TGTTGGGGCTCCTCAGGTTC-3’
Cyclin E1	Forward Reverse	5’-CCACACCTGACAAAGAAGATGATGAC-3’ 5’-GAGCCTCTGGATGGTGCAATAAT-3’
CDK4	Forward Reverse	5’-GAGGGGGCCTCTCTAGCTT-3’ 5’-CACGGGTGTAAGTGCCATCT-3’
c-myc	Forward Reverse	5’-GCCACGTCTCCACACATCAG-3’ 5’-TGGTGCATTTTCGGTTGTTG-3’
Bax	Forward Reverse	5’-GAAGCCGGTGGTGGAGAA-3’ 5’-GCTTGGAGTTGGGCTGGTG-3’
Bcl-2	Forward Reverse	5’- GAAGCAGGTAATGGAGCAAGGA-3’ 5’-GAAGCGTAGTTGTTGAGATGCG-3’
MMP-2	Forward Reverse	5’-GAGTGCATGAACCAACCAGC-3’ 5’-AAACTTGCAGGGCTGTCCTT-3’
MMP-9	Forward Reverse	5’-TCTATGGTCCTCGCCCTGAA-3’ 5’-TTGTATCCGGCAAACTGGCT-3’
Survivin	Forward Reverse	5’-ATTTGAATCGCGGGACCC-3’ 5’-GAGAAAGGGCTGCCAGGC-3’
GAPDH	Forward Reverse	5’-ACTTTGGTATCGTGGAAGGACTCAT-3’ 5’-GTTTTTCTAGACGGCAGGTCAGG-3’

### Western-blot analysis

Total protein was extracted by Lysis Buffer. Lysates were incubated on ice for 30 min, followed by centrifugation at 16,000 × g at 4°C for 10 min to remove cellular debris, then the supernatants were collected. A bicinchoninic acid assay (BCA) was performed to quantify protein concentrations. Protein samples (40 μg/lane) were separated on a 0% SDS-PAGE gel and subsequently transferred to polyvinylidene difluoride (PVDF) membranes. The membranes were blocked in a solution of Tris buffered saline containing 0.1% Tween-20 and 5% nonfat milk for 2h at room temperature. Then incubated with primary antibodies at 4°C overnight. After washing with PBST buffer (10 mM phosphate buffer, 40mM NaCl, 2.7mM KCl and 0.05% Tween 20, pH 7.4), membranes were incubated with horseradish peroxidase-conjugated secondary antibodies for 2 h at room temperature. Finally, membranes were treated with an enhanced chemiluminescence substrate. GAPDH was used as the loading control.

### Luciferase assay

To evaluate the Wnt/β-catenin signaling pathway activity, the TOP-flash/FOP-flash firefly luciferase and pTK-RL Renilla luciferase constructs were applied to measure the activation of the Wnt/β-catenin pathway after baicalein treatment. Briefly, 143B and MG-63 cells grown on 24-well plates at a density of 2 × 10^5^ cells/well. Osteosarcoma cells were co-transfected with TCF-luciferase reporter either wildtype (TOP-flash) or mutant (FOP-flash) along with a control TK-Renilla plasmid. The reporter vector were transfected into 143B and MG-63 cells using Lipofectamine™ 2000 transfection Reagent (Invitrogen, Carlsbad, CA, USA) for 8 h according to the manufacturer’s protocol. Subsuquently, the osteosarcoma cells were treated with different doses of baicelain for 48 h, then the cells were lysed and the luciferase activity was measured using the Dual-Luciferase Assay System (Promega, Madison, WI, USA) and a Veritas Microplate Luminometer (Turner Biosystems, Promega, Madison, WI, USA). Fireflyluciferase activity was normalized to activity of Renilla Luciferase. For each experiment, the luciferase assay was performed three times.

### 143B tumor xenograft model in nude mice

Male BALB/c-nu/nu nude mice were purchased from Beijing HFK Experiment Animal Center (Beijing, China) and housed in laminar flow cabinets under specific pathogen-free conditions with food and water ad libitum. The study protocol was approved by the Experimental Animal Care Committee of Renmin Hospital of Wuhan University and conforms with the provisions of the Declaration of Helsinki. All surgeries were performed under sodium pento–barbital anesthesia (Sigma, St. Louis, MO, USA), and all efforts were made to minimize suffering. Male nude mice were introduced to establish xenograft tumor model of 143B cells as previously described [26]. 6 nude mice were randomly selected from 24 nude mice as healthy mice group named (a) before subcutaneous injection of the 143B cells. Subsequently, 143B cells (5 × 10^6^ cells in 0.2 mL of serum-free medium) were subcutaneously injected into the armpit of the remaining 8 nude mice. Seven days after implantation, the nude mice were randomly divided into 3 groups with 6 mice each group: (b) vehicle: equivalent amount of DMSO in Normal Saline (NS); (c) lower concentration group: 50 mg/kg of baicalein; (d) higher concentration group: 100 mg/kg of baicalein. NS or baiclein were injected intraperitoneally (i.p.) once every other day for 5 weeks. Then, all animals were sacrificed by dislocation, and the tumors, heart, liver, spleen, lung, and kidney were collected and measured. During animal experiment period the mice were examined daily for toxicity/mortality relevant to treatment, and the tumor was measured with a caliper once a week for up to 5 weeks. Mice body weight and mice survival (at days 100, in another experiment) were also recorded. The tumor volume (V) (in mm^3^) was calculated according to the formula: V = W^2^ × L/2, where W and L represent the shortest and longest diameters, respectively. And the curve of tumor growth was depicted 6 weeks after inoculation.

### HE staining

As for hematoxylin and eosin (HE) staining, the extracted tissues were fixed with 4% paraformaldehyde for 24 h, dehydrated with an ethanol gradient, and embedded in paraffin. The paraffin tissues were cut into 4-μm-thick sections and then stained with HE.

### Immunohistochemistry and TUNEL assay

The mice were sacrificed by euthanasia. The tissues were fixed with 4% paraformaldehyde for 24 h, dehydrated, and coated with wax. Tissue sections were sliced to 4-μm-thick sections and dyed with the primary antibody. Photographes were taken by the Olympus microscope (×400). Immunostaining intensity was estimated quantitatively according to signal intensity and distribution. TUNEL assay was performed to detect apoptosis of tumor tissues. An in situ apoptosis detection kit (Roche Diagnostics) was used to detect apoptotic cells in paraffin tumor tissue sections. The sections were observed under the Olympus microscope (×400). The positive cells were identified, counted (ten random fields per slides), and analyzed.

### Statistical analysis

Statistical analyses were performed using the SPSS 6.0 statistical software package (SPSS, Inc., Chicago, IL, USA). Each experiment was repeated at least three times. All data are expressed as the mean ± standard deviation (SD). The Student’s t-test was used to compare the means of 2 groups. Where more than 3 means were compared, one-way ANOVA followed by multiple comparisons among the means was used. The Kaplan–Meier method was used to calculate the survival curve, and Log-Rank test to determine statistical significance. *P<0.05* was considered statistically significant.

## References

[1] Isakoff MS, Bielack SS, Meltzer P, Gorlick R (2015). Osteosarcoma: Current Treatment and a Collaborative Pathway to Success. J Clin Oncol.

[2] Janeway KA, Grier HE (2010). Sequelae of osteosarcoma medical therapy: a review of rare acute toxicities and late effects. Lancet Oncol.

[3] Vijayamurugan N, Bakhshi S (2014). Review of management issues in relapsed osteosarcoma. Expert Rev AnticanC.

[4] Luetke A, Meyers PA, Lewis I, Juergens H (2014). Osteosarcoma treatment - where do we stand? A state of the art review. Cancer Treat Rev.

[5] Maran A, Dadsetan M, Buenz CM, Shogren KL, Lu L, Yaszemski MJ (2013). Hydrogel-PLGA delivery system prolongs 2-methoxyestradiol-mediated anti-tumor effects in osteosarcoma cells. J Biomed Mater Res A.

[6] Huang Y, Zhao K, Hu Y, Zhou Y, Luo X, Li X, Wei L, Li Z, You Q, Guo Q, Lu N (2016). Wogonoside inhibits angiogenesis in breast cancer via suppressing Wnt/β-catenin pathway. Mol Carcinog.

[7] Teiten MH, Gaascht F, Dicato M, Diederich M (2012). Targeting the wingless signaling pathway with natural compounds as chemopreventive or chemotherapeutic agents. Curr Pharm Biotechnol.

[8] Lin CH, Ji T, Chen C, Hoang BH https://doi.org/10.1007/978-3-319-04843-7_2.

[9] Anastas JN, Moon RT (2013). WNT signalling pathways as therapeutic targets in cancer. Nat Rev Cancer.

[10] Takebe N, Miele L, Harris PJ, Jeong W, Bando H, Kahn M, Yang SX, Ivy SP (2015). Targeting Notch, Hedgehog, and Wnt pathways in cancer stem cells: clinical update. Nat Rev Clin Oncol.

[11] Cai Y, Cai T, Chen Y (2014). Wnt pathway in osteosarcoma, from oncogenic to therapeutic. J Cell Biochem.

[12] Ma Y, Zhu B, Liu X, Yu H, Yong L, Liu X, Shao J, Liu Z (2015). Inhibition of oleandrin on the proliferation show and invasion of osteosarcoma cells *in vitro* by suppressing Wnt/β-catenin signaling pathway. J Exp Clin Cancer Res.

[13] Chen C, Zhao M, Tian A, Zhang X, Yao Z, Ma X (2015). Aberrant activation of Wnt/β-catenin signaling drives proliferation of bone sarcoma cells. Oncotarget.

[14] Wunder JS, Nielsen TO, Maki RG, O’Sullivan B, Alman BA (2007). Opportunities for improving the therapeutic ratio for patients with sarcoma. Lancet Oncol.

[15] Martins-Neves SR, Paiva-Oliveira DI, Wijers-Koster PM, Abrunhosa AJ, Fontes-Ribeiro C, Bovée JV, Cleton-Jansen AM, Gomes CM (2016). Chemotherapy induces stemness in osteosarcoma cells through activation of Wnt/β-catenin signaling. Cancer Lett.

[16] Lu XS, Qiao YB, Li Y, Yang B, Chen MB, Xing CG (2017). Preclinical study of cinobufagin as a promising anti-colorectal cancer agent. Oncotarget.

[17] XH Z. YX L, M J, JS H, M Z, SP J and AM L. Oridonin inhibits tumor growth in glioma by inducing cell cycle arrest and apoptosis. Cell Mol Biol. 2014; 60:29–36 25553351

[18] Wang CY, Bai XY, Wang CH (2014). Traditional Chinese medicine: a treasured natural resource of anticancer drug research and development. Am J Chin Med.

[19] Xie T, Ren HY, Lin HQ, Mao JP, Zhu T, Wang SD, Ye ZM (2016). Sinomenine prevents metastasis of human osteosarcoma cells via S phase arrest and suppression of tumor-related neovascularization and osteolysis through the CXCR4-STAT3 pathway. Int J Oncol.

[20] Zhang Y, Fox JT, Park YU, Elliott G, Rai G, Cai M, Sakamuru S, Huang R, Xia M, Lee K, Jeon MH, Mathew BP, Park HD (2016). A Novel Chemotherapeutic Agent to Treat Tumors with DNA Mismatch Repair Deficiencies. Cancer Res.

[21] Wang Y, Han E, Xing Q, Yan J, Arrington A, Wang C, Tully D, Kowolik CM, Lu DM, Frankel PH, Zhai J, Wen W, Horne D (2015). Baicalein upregulates DDIT4 expression which mediates mTOR inhibition and growth inhibition in cancer cells. Cancer Lett.

[22] Kim SD, Lee YJ, Baik JS, Han JY, Lee CG, Heo K, Park YS, Kim JS, Ji HD, Park SI, Rhee MH, Yang K (2014). Baicalein inhibits agonist- and tumor cell-induced platelet aggregation while suppressing pulmonary tumor metastasis via cAMP-mediated VASP phosphorylation along with impaired MAPKs and PI3K-Akt activation. Biochem Pharmacol.

[23] Ma X, Yan W, Dai Z, Gao X, Ma Y, Xu Q, Jiang J, Zhang S (2016). Baicalein suppresses metastasis of breast cancer cells by inhibiting EMT via downregulation of SATB1 and Wnt/β-catenin pathway. Drug Des Devel Ther.

[24] Sheppard KE, McArthur GA (2013). The cell-cycle regulator CDK4: an emerging therapeutic target in melanoma. Clin Cancer Res.

[25] Malumbres M, Barbacid M (2009). Cell cycle, CDKs and cancer: a changing paradigm. Nat Rev Cancer.

[26] Yu L, Fan Z, Fang S, Yang J, Gao T, Simões BM, Eyre R, Guo W, Clarke RB (2016). Cisplatin selects for stem-like cells in osteosarcoma by activating Notch signaling. Oncotarget.

[27] Hattinger CM, Fanelli M, Tavanti E, Vella S, Ferrari S, Picci P, Serra M (2015). Advances in emerging drugs for osteosarcoma. Expert Opin Emerg Drugs.

[28] Mu J, Liu T, Jiang L, Wu X, Cao Y, Li M, Dong Q, Liu Y, Xu H (2016). The Traditional Chinese Medicine Baicalein Potently Inhibits Gastric Cancer Cells. J Cancer.

[29] Liu TY, Gong W, Tan ZJ, Lu W, Wu XS, Weng H, Ding Q, Shu YJ, Bao RF, Cao Y, Wang XA, Zhang F, Li HF (2015). Baicalein inhibits progression of gallbladder cancer cells by downregulating ZFX. PLoS One.

[30] Zheng YH, Yin LH, Grahn TH, Ye AF, Zhao YR, Zhang QY (2014). Anticancer effects of baicalein on hepatocellular carcinoma cells. Phytother Res.

[31] Wang W, Xi M, Duan X, Wang Y, Kong F (2015). Delivery of baicalein and paclitaxel using self-assembled nanoparticles: synergistic antitumor effect *in vitro* and *in vivo*. Int J Nanomedicine.

[32] Wang CZ, Zhang CF, Chen L, Anderson S, Lu F, Yuan CS (2015). Colon cancer chemopreventive effects of baicalein, an active enteric microbiome metabolite from baicalin. Int J Oncol.

[33] Wang L, Ling Y, Chen Y, Li CL, Feng F, You QD, Lu N, Guo QL (2010). Flavonoid baicalein suppresses adhesion, migration and invasion of MDA-MB-231 human breast cancer cells. Cancer Lett.

[34] Chen H, Gao Y, Wu J, Chen Y, Chen B, Hu J, Zhou J (2014). Exploring therapeutic potentials of baicalin and its aglycone baicalein for hematological malignancies. Cancer Lett.

[35] Li-Weber M (2013). Targeting apoptosis pathways in cancer by Chinese medicine. Cancer Lett.

[36] Slusarz A, Shenouda NS, Sakla MS, Drenkhahn SK, Narula AS, MacDonald RS, Besch-Williford CL, Lubahn DB (2010). Common botanical compounds inhibit the hedgehog signaling pathway in prostate cancer. Cancer Res.

[37] Zhou RT, He M, Yu Z, Liang Y, Nie Y, Tai S, Teng CB (2017). Baicalein inhibits pancreatic cancer cell proliferation and invasion via suppression of NEDD9 expression and its downstream Akt and ERK signaling pathways. Oncotarget.

[38] Zhang S, Bao Y, Ju X, Li K, Shang H, Ha L, Qian Y, Zou L, Sun X, Li J, Wang Q, Fan Q (2015). BA-j as a novel CDK1 inhibitor selectively induces apoptosis in cancer cells by regulating ROS. Sci Rep.

[39] Gulappa T, Reddy RS, Suman S, Nyakeriga AM, Damodaran C (2013). Molecular interplay between cdk4 and p21 dictates G0/G1 cell cycle arrest in prostate cancer cells. Cancer Lett.

[40] Bie B, Sun J, Li J, Guo Y, Jiang W, Huang C, Yang J, Li Z (2017). Baicalein, a Natural Anti-Cancer Compound, Alters MicroRNA Expression Profiles in Bel-7402 Human Hepatocellular Carcinoma Cells. Cell Physiol Biochem.

[41] Tanaka M, Setoguchi T, Hirotsu M, Gao H, Sasaki H, Matsunoshita Y, Komiya S (2009). Inhibition of Notch pathway prevents osteosarcoma growth by cell cycle regulation. Br J Cancer.

[42] Xu W, Wang B, Yang M, Zhang Y, Xu Z, Yang Y, Cao H, Tao L (2017). Tebufenozide induces G1/S cell cycle arrest and apoptosis in human cells. Environ Toxicol Pharmacol.

[43] Kim SL, Kim SH, Trang KT, Kim IH, Lee SO, Lee ST, Kim DG, Kang SB, Kim SW (2013). Synergistic antitumor effect of 5-fluorouracil in combination with parthenolide in human colorectal cancer. Cancer Lett.

[44] Li X, Zhu F, Jiang J, Sun C, Wang X, Shen M, Tian R, Shi C, Xu M, Peng F, Guo X, Wang M, Qin R (2015). Synergistic antitumor activity of withaferin A combined with oxaliplatin triggers reactive oxygen species-mediated inactivation of the PI3K/AKT pathway in human pancreatic cancer cells. Cancer Lett.

[45] Kiraz Y, Adan A, Kartal Yandim M, Baran Y (2016). Major apoptotic mechanisms and genes involved in apoptosis. Tumour Biol.

[46] Signore M, Ricci-Vitiani L, De Maria R (2013). Targeting apoptosis pathways in cancer stem cells. Cancer Lett.

[47] Ganguly KK, Pal S, Moulik S, Chatterjee A. Kirat Kumar Ganguly, Sekhar Pal, Shuvojit Moulik & Amitava Chatterjee. Integrins and metastasis. Cell Adhes Migr. 2013;7:251–61 10.4161/cam.23840 PMC3711990 23563505

[48] Herszényi L, Hritz I, Lakatos G, Varga MZ, Tulassay Z (2012). The behavior of matrix metalloproteinases and their inhibitors in colorectal cancer. Int J Mol Sci.

[49] Jang GB, Kim JY, Cho SD, Park KS, Jung JY, Lee HY, Hong IS, Nam JS (2015). Blockade of Wnt/β-catenin signaling suppresses breast cancer metastasis by inhibiting CSC-like phenotype. Sci Rep.

[50] Klaus A, Birchmeier W (2008). Wnt signalling and its impact on development and cancer. Nat Rev Cancer.

[51] Watson AL, Rahrmann EP, Moriarity BS, Choi K, Conboy CB, Greeley AD, Halfond AL, Anderson LK, Wahl BR, Keng VW, Rizzardi AE, Forster CL, Collins MH (2013). Canonical Wnt/β-catenin signaling drives human schwann cell transformation, progression, and tumor maintenance. Cancer Discov.

[52] Radulescu S, Ridgway RA, Cordero J, Athineos D, Salgueiro P, Poulsom R, Neumann J, Jung A, Patel S, Woodgett J, Barker N, Pritchard DM, Oien K, Sansom OJ (2013). Acute WNT signalling activation perturbs differentiation within the adult stomach and rapidly leads to tumour formation. Oncogene.

[53] Tian J, He H, Lei G (2014). Wnt/β-catenin pathway in bone cancers. Tumour Biol.

[54] He N, Zhang Z (2015). Baicalein suppresses the viability of MG-63 osteosarcoma cells through inhibiting c-MYC expression via Wnt signaling pathway. Mol Cell Biochem.

[55] Zhang RX, Li Y, Tian DD, Liu Y, Nian W, Zou X, Chen QZ, Zhou LY, Deng ZL, He BC (2016). Ursolic acid inhibits proliferation and induces apoptosis by inactivating Wnt/β-catenin signaling in human osteosarcoma cells. Int J Oncol.

[56] Lv Z, Wang C, Yuan T, Liu Y, Song T, Liu Y, Chen C, Yang M, Tang Z, Shi Q, Weng Y (2014). Bone morphogenetic protein 9 regulates tumor growth of osteosarcoma cells through the Wnt/β-catenin pathway. Oncol Rep.

[57] Wang DZ, Gao JF, Jing SF, Wei H, Huang XY, Li CD (2015). Antitumor Effect of Docetaxel in Osteosarcoma by the Inhibition of Wnt Signal Channel. Drug Res (Stuttg).

[58] Goldstein SD, Trucco M, Bautista Guzman W, Hayashi M, Loeb DM (2016). A monoclonal antibody against the Wnt signaling inhibitor dickkopf-1 inhibits osteosarcoma metastasis in a preclinical model. Oncotarget.

[59] Tang QL, Zhao ZQ, Li JC, Liang Y, Yin JQ, Zou CY, Xie XB, Zeng YX, Shen JN, Kang T, Wang J (2011). Salinomycin inhibits osteosarcoma by targeting its tumor stem cells. Cancer Lett.

[60] Liu X, Liu S, Chen J, He L, Meng X, Liu S (2016). Baicalein suppresses the proliferation of acute T-lymphoblastic leukemia Jurkat cells by inhibiting the Wnt/β-catenin signaling. Ann Hematol.

